# Medical borderlands: engineering the body with plastic surgery and hormonal therapies in Brazil

**DOI:** 10.1080/13648470.2014.918933

**Published:** 2014-08-30

**Authors:** Alexander Edmonds, Emilia Sanabria

**Affiliations:** ^a^ University of Edinburgh, Edinburgh, UK; ^b^ Ecole normale supérieure de Lyon, Laboratoire Triangle & INSERM, 15 Parvis René Descartes, Lyon, France

**Keywords:** plastic surgery, hormonal therapies, Brazil, gender and sexuality, reproductive health

## Abstract

This paper explores medical borderlands where health and enhancement practices are entangled. It draws on fieldwork carried out in the context of two distinct research projects in Brazil on plastic surgery and sex hormone therapies. These two therapies have significant clinical overlap. Both are made available in private and public healthcare in ways that reveal the class dynamics underlying Brazilian medicine. They also have an important experimental dimension rooted in Brazil's regulatory context and societal expectations placed on medicine as a means for managing women's reproductive and sexual health. Off-label and experimental medical use of these treatments is linked to experimental *social* use: how women adopt them to respond to the pressures, anxieties and aspirations of work and intimate life. The paper argues that these experimental techniques are becoming morally authorized as routine management of women's health, integrated into mainstream Ob-Gyn healthcare, and subtly blurred with practices of *cuidar-se* (self-care) seen in Brazil as essential for modern femininity.

## Introduction

Medical enhancement has become an urgent social issue in part because it sometimes – and a lot depends on how often – works. The athlete takes steroids, at considerable risk, because they really do enhance performance. And as medical enhancement becomes more efficacious (however defined), it finds new users: China and Brazil are now respectively the world's third and second largest markets for cosmetic surgery, while in many developing countries ‘prescription’ drugs are used to boost sexual or mental performance (Sanabria forthcoming). As medical enhancement spreads, it becomes harder to evaluate its health and ethical implications. While technologies such as genetic engineering have provoked weighty ethical questions about ‘tampering with nature’ or ‘playing God’ (Parens 1998), such questions can also distract us from the problems raised by more ordinary forms of medical self-regulation and self-optimization that occur within routine healthcare.

This paper explores medical borderlands where health and enhancement practices are subtly entangled. It is based on ethnographic fieldwork in Brazilian cities, carried out in the context of two distinct research projects: on the uses of plastic surgery in Rio de Janeiro (Edmonds) and sex hormone therapies in Salvador da Bahia (Sanabria), primarily by women. During fieldwork we each interviewed healthcare practitioners (mostly surgeons, psychologists, endocrinologists, GPs, ObGyns, and nurses), observed clinical practices, and conducted ethnographic work with patient-consumers that took us into social contexts not directly tied to medical care.[Fn en0001]

Cosmetic surgery and sex hormones are very different kinds of therapies that target different aspects of the body, health and well-being.[Fn en0002] But in Brazil they have important areas of clinical overlap as a means of intervening in, or optimizing, women's reproductive and sexual health and well-being. Hormonal contraceptives are among the most widely prescribed drugs in pharmaceutical history. In Brazil, they are widely available in a range of combinations and modes of delivery. They also have controversial rationales not limited to contraception or treatment of menopausal symptoms, such as regulating menstruation, body shape, or sexual and social performance. Such off label uses are often blurred with more acceptable indications such as birth control. We thus use the local term *hormônio* to include the overlapping indications and experimental uses of these drugs in Brazil. Globally, the availability of cosmetic surgery is more limited and is generally considered a form of enhancement. But in Brazil, the full range of procedures – including breast and abdominal surgeries, liposuction and ‘*lipoescultura*’ (reinjecting body fat), nose jobs, and face lifts – have become more widely available in recent decades, and are often presented as an essential means of managing women's reproductive, sexual and psychological health. Following local usage, we refer to cosmetic surgeries as ‘*plástica*.’

We bring these two cases together to explore how they have become normalized within a two-tier healthcare system and used experimentally to manage women's reproductive and sexual lives. *Plástica* and *hormônio* are available in both private and public healthcare in ways that reveal the class dynamics underlying Brazilian medicine. Moreover, both therapies have an important experimental dimension, which we link to Brazil's regulatory context and societal expectations placed on medicine as a means for managing women's health.

We argue these therapies are sometimes used with the intent of engineering the sexual and reproductive body from the inside out. We further argue that these therapies are becoming morally authorized as routine ways of managing women's health. They are often merged with cosmetic and hygienic regimes of *cuidar-se* (self-care) seen as essential for modern femininity. Pharmacies, beauty parlors, gyms, health food shops, private medical practices, and pseudo-medical cosmetic services abound in Brazilian cities. The patients we encountered viewed *hormônio* and *plástica* as integral parts of ordinary regimes of *cuidar-se* that included work-outs and weight-management, having manicures, hair straightening, cultivating tan lines, or the ‘Brazilian’ body wax. The distinctions between cosmetic, hygienic, erotic and health practices are thus often blurred – as are the lines between necessary medical interventions and those with more diffuse rationales of boosting *auto-estima* (self-esteem) or managing life's exigencies.

While experimental uses of *hormônio* and *plástica* pose risks to health, they also raise ethical problems that do not readily fit the conceptual frameworks bioethicists have often used to discuss medical enhancement (see Parens 1998 and Gordijn and Chadwick 2008). The very ordinariness of contraceptives can mask their more experimental uses in boosting mood, controlling weight or regulating sexual or social life. And while *plástica* may seem to be a more obvious case of medical enhancement, its rationale of enhancement is subtly minimized as it is practiced in public hospitals or integrated with mainstream healthcare. Some Brazilian doctors and patients view both of these therapies as a means of everyday self-care, suggesting that they are not always locally considered ‘enhancement’ in the first place. Concerns about ‘tampering with nature’ – rooted in liberal political ideas about bodily autonomy – do not always translate well to other ethical contexts (Edmonds 2010). Brazilian folk and medical ontologies see the body as considerably *plastic* – amenable to change and improvement (Rohden 2001, Sanabria 2013).

We suggest a different critical approach, one that focuses on both the experimental *medical uses* of these therapies as well as what can be called their experimental *social uses*. We explore how these technologies are adopted to respond to pressures, anxieties and aspirations arising out of work and intimate life. These uses often diverge from official medical discourse or public health agendas, although they can receive the tacit support and encouragement of doctors. We discuss, for example, how *plástica* and *hormônio* are used for non-medical purposes such as modulating affects or physical strength at work, opening social doors, emulating *Playboy* models, boosting desire, or protecting a marriage – uses that reveal gendered tensions in the medical and social norms of modern femininity. *Plástica* and *hormônio* are integrated into a management regime that makes ‘normal’ reproduction (menstruation, birth, breast feeding and menopause) into risky processes requiring ‘aesthetic’ as well as sexual management. Aesthetic goals are morally authorized as health goals while experimental re-tooling is rendered as necessary, routine or even pleasurable self-care.

How do medical technologies reflect aspirations for social inclusion or mobility? How do experimental, aesthetic or lifestyle uses of medicine become legitimated and integrated with more established forms of healthcare? In responding to these questions our goal is to develop a critical approach to experimental techniques practiced in the ‘borderlands’ between health-oriented medicine and aesthetic, sexual and psychological self-care.

## Therapies in search of disease

In Brazil, *hormônio* and *plástica* now have medical rationales as a means of intervening in psychological, sexual and reproductive health in ‘normal’ patients (by altering endocrinological systems, anatomy, and body-image). Historically, however, there has been considerable medical and social uncertainty as to the appropriate use of both of these therapies. We briefly trace their origins as experimental technologies and contextualize their shifting medical indications.

Historian Oudshoorn (1994) calls hormonal therapies ‘drugs looking for diseases.’ Likewise for plastic surgery, the possibility of reasonably safe aesthetic operations preceded the ‘discovery’ of the disorders they would heal. Both *plástica* and *hormônio* were products of medical advances that held out for some pioneers' enormous therapeutic – and financial – potential. Both also predated the medical indication they eventually ‘found.’ Sex hormones were first marketed in the late 1950s as treatments for menstrual disorders; their contraceptive properties were presented as ‘side effects’ to gauge market acceptability for a controversial new drug (Marks 2001, 5). Today, birth control is the main approved indication for hormonal contraceptives, although they have a range of off-label uses including alleviation of menstrual cramps, menstrual suppression, regulating the cycle, libido regulation, ‘lifestyle’ indications (see Watkins 2012), and in Brazil, controversial ‘aesthetic’ uses such as weight control and improving skin texture and muscle tone.[Fn en0003] Since their invention, the rationale for the use of sex hormones has been open to negotiation and subject to controversy.

Contraceptives in Brazil come in a multitude of delivery systems, dosages and modes of administration (Sanabria, forthcoming). In addition to variations in oral forms (brand-name, national copies or generics, combined or minipills, second, third or fourth generation, etc.), sex hormones may be implanted subdermally, absorbed through the skin (via transdermal patches or gels), the vagina (via a vaginal ‘ring’) or the uterus (via an intra-uterine hormone-releasing ‘system’) or injected (monthly or trimesterly). This multiplicity reflects the search for long-acting contraceptive methods for the developing world, in which Brazilian medical institutions have played a significant role.[Fn en0004]

Doctors also have considerable leeway to experiment with new regimens. This is particularly true for hormonal implants developed in Brazil where standardized, brand-name implants such as *Implanon* are rare. The bulk of implants on the market are produced by *farmácias de manipulação* (compounding pharmacies) in small-scale laboratories[Fn en0005] and contain combinations of levonongesterel, estradiol, gestrinone, elcometrin and testosterone. Although these implants are presented as state-of-the art treatments, their production is remarkably low-tech and labor-intensive. In one such pharmacy, Sanabria observed lab technicians manually crafting implants from silicon tubing and vats of imported, powdered *hormônio*. Implants have a range of blurred indications: as long-acting contraception, to ‘control’ pre-menstrual symptoms or ‘treat’ endometriosis by suppressing menstruation, and as hormone-replacement for menopausal symptoms. These treatments are not all clinically trialed, unlike those produced by transnational pharmaceutical corporations. The only published data come from observational studies of women, many of them conducted in Brazil, with loose ethical oversight. One woman Sanabria interviewed in 2013 explained that her gynecologist had asked her to perform clinical exams (at her own expense), including cardiovascular and cholesterol analyses. Upon finding out online that her gynecologist had published a study in which she was likely included, she became outraged by the risks she had been subjected to as – in her words – ‘a guinea pig’ in an experiment.

In one of the more radical examples of experimentation with sex hormones, *travestis* (transsexuals) use over-the-counter contraceptives to feminize their bodies, often in combination with injected liquid silicone and, more rarely, medical *plástica* (Sanabria 2013; Kulick 1998; Edmonds 2010). These techniques ‘soften’ facial structure and change fat distribution to emulate national erotic ideals. But women also, and in much greater numbers, use sex hormones in pursuit of sexual and ‘aesthetic’ effects, most notably with testosterone (T). Testosterone therapy regularly makes the headlines in Brazil for its benefits during work-outs or to boost energy, *vontade* (will/desire), and *disposição* (disposition/desire). Marketing and health care practitioners present T as a kind of aphrodisiac, although women's descriptions of their experiences are more ambivalent. While observing the routine insertion or removal of hormonal implants in a Bahian clinic, Sanabria noted the commentaries exchanged regarding the small clump of hair-growth that appears at the place of implantation, usually the *bunda* (‘bum’). The specialized nurse viewed this tiny clump of abnormally long, thick hair as visual ‘evidence’ that testosterone *really* works.

Although the doctors whom Sanabria observed prescribing implants usually referred to testosterone as *dar fogo* (to give fire, an explicitly sexual metaphor), patients tended to emphasize wider dispositional changes. Cleonice is in her mid-40s and married to an architect from the South of Brazil. Sanabria met her in a clinic where she was having her implants renewed, which she had been using for over a decade. Cleonice had also had several ‘minor’ – in her words – *plásticas* (to the eyelids, nose, and facelift). ‘*Gosto de me cuidar* (I like caring for myself),’ she explained, ‘and hormones help me feel good, they help the body, the skin. It improves your *performance* (in English), you have more *disposition*.’ While disposition sometimes refers to sexual interest, Cleonice emphasized that *hormônio* gives her ‘energy to face her day-to-day, with more *vontade* (will).’ Besides the increased desire it is said to afford, women explained that T gives them energy to see or perceive differently and to be more operational in a male world. In 2008 a series of press articles described the increasing use of testosterone implants by Brazilian women in *academias* (gyms). Referred to as *mulheres chipadas* (women with a microchip), these women said that they build more muscle mass, lose subdermal fat more rapidly, and optimize their workout with T. One photographer explained to Sanabria that her gynecologist had added a T rod to give her more *tesão* (sexual desire). She added that, thanks to T, her camera boxes felt much lighter and she had more energy for her demanding job and for managing her work/life balance.

While they are not endorsed by all users, drug effects are conditioned by patient expectations (Moerman and Jonas 2002). Given the absence of clinical trials assessing such experimental uses of contraceptive implants, it is difficult to separate specific ‘drug effects’ from the larger medical and social contexts that make them efficacious. The sexual effects of hormones subtly mingle with lifestyle and ‘aesthetic effects’ (discussed below) such as weight loss, diminishing cellulite, firming muscles, and improving skin texture.

In contrast to the banality of the pill, cosmetic surgery is often considered an exceptional – sometimes even a dangerous – treatment. Yet, reconstructive procedures that today are perceived as medically uncontroversial, such as correction of a cleft palate, were once labeled ‘cosmetic’ (Gilman 1999). Cosmetic orthodontics is still classified as ‘cosmetic’ but has also become routinized to the point where denying it to a child is considered – in the US at least – cruel or irresponsible. We argue that cosmetic surgery in Brazil is moving towards routinization and becoming an integral part of managing passage through the female lifecycle.

While doctors had previously experimented with cosmetic procedures, it was only the consolidation of technical skills through the repairing of the shattered faces of First World War veterans that raised the possibility of effective cosmetic surgery. Yet social and medical attitudes at the time viewed the use of surgery for exclusively aesthetic aims as frivolous (Gilman 1999). Euro-American society caught up to the therapeutic possibilities of cosmetic surgery in part due to the popularization of psychoanalytic research pointing to the emotional significance of appearance (Haiken 1997). Brazilian surgeons have successfully advanced a radical ‘psychotherapeutic rationale’ for cosmetic surgery, even justifying its provision in public hospitals. The national healthcare system (*Sistema único de saúde* or SUS) does not directly authorize cosmetic surgery (with the exception of a few procedures). However, some public hospitals offer cosmetic surgeries, which like all public sector healthcare is free, in order to provide ‘scientific training’ to surgical residents. Some chief public surgeons have supported this logic by invoking the universal ‘health’ benefits of cosmetic surgery, which they believe should be available to rich and poor alike (Edmonds 2009, 2010).

Surgical residents from many countries travel to train in Brazil's plastic surgery wards, drawn by opportunities for hands-on practice in cosmetic techniques (Edmonds 2011). One European resident said he had performed 96 surgeries during his third year of residency, of which 90% were cosmetic. Another European resident, surprised to observe two major aesthetic surgeries being performed simultaneously in the same room, remarked that Brazilian surgeons are highly skilled and that complication rates are low.[Fn en0006] In fact patients may be more at risk in some private clinics, which unlike public hospitals do not have life support systems. High demand and the busy schedules of teaching hospitals have contributed, some plastic surgeons boast, to a specifically Brazilian spirit of “creativity” and “innovation” (Edmonds 2010).

Other surgical techniques were developed in response to Brazil's *mestiço* population and beauty norms, according to surgeons whom Edmonds interviewed. Surgeons explained that patients with African ancestry have a higher risk of forming thick keloid scars, but that due to extensive racial mixing ancestry is not always visible in phenotype. Thus, before deciding whether an aesthetic breast reduction has a good risk-benefit ratio, a small ‘test’ incision between the patient's breasts is made to see whether she will develop such scars. Surgeons also use cosmetic techniques to emulate the ostensibly ‘good’ effects of what they call ‘miscegenation’ – such as ‘body contouring’ that redistributes fat from the waist to the hips. But they also say surgery can correct negative effects attributed to racial mixing, for example by thinning the nose in order to ‘harmonize’ it with the overall appearance of a patient who identifies as *branca* (white) or *morena* (brown). Such experimental ‘aesthetic indications’ are legitimated by the fact that cosmetic surgeries are performed in public hospitals, or offered as interventions in women's reproductive health (Edmonds 2013a).

Patients are often willing to try experimental techniques, combine surgeries, or have repeated *retoques* (touch-ups). The injection of industrial-grade liquid silicone (considered unacceptably risky and now banned) is usually associated with the transformation of transvestites in Brazil and other countries. However, Brazilian women have also had liquid silicone injected by doctors or have experimented with this practice themselves, some coming to public hospitals for corrections. Other patients opt for surgery at the prompting of doctors to correct flaws they had not perceived themselves. Raquel went to a public hospital for cosmetic breast surgery, but given the long waiting time, chose a nose job instead, her second preference. Many patients remain uninformed about the risks of complications, which are rarely discussed and often downplayed for both hormonal treatments and *plástica*.

Some experimental uses of these therapies occur at the fringes of ‘mainstream’ medical practice with little medical supervision. With both *plástica* and *hormônio* we observed patients actively seeking out practitioners willing to perform interventions they had heard about in *telenovelas* or through acquaintances and relatives. Brazilian plastic surgery and gynecological wards, we observed, are highly social places where information about procedures, hospitals, and sympathetic doctors are traded *boca a boca* (word of mouth). We now explore further how these therapies are situated within women's healthcare by discussing the class dynamics in Brazilian medical institutions.

## The class biopolitics of *plástica* and *hormônio*

Post-dictatorship Brazil (1985–) has witnessed rapid, often contradictory changes in healthcare (Biehl 2005). While social movements succeeded in enshrining the right to healthcare in the 1988 constitution, the middle classes opted out of (public) healthcare and turned to private sector services accessed through relatively cheap health insurance – a development Faveret and Oliveira (1989) refer to as ‘excluding universalism.’

Brazil now has a highly differentiated healthcare system split between a public and a private sector, to which roughly 25% of the population has access. Some private clinics are shabby but free of the long public sector queues, while top-end institutions have the hallmarks of luxury and glamour. The equally heterogeneous public sector is often perceived as offering lower quality care. Although they are often represented as separate, doctors, patients and protocols travel between the public and private sectors (Sanabria 2010).

Both *plástica* and *hormônio* are offered in private and public clinics and hence target users from a wide range of socioeconomic backgrounds. Oral contraceptives have been widely available in Brazil since the 1970s when the state reversed its pro-natalist position. But there continue to be important class differences in prescription patterns for hormonal contraceptives. Rose (2007) and Rose and Rabinow (2006) have distinguished between an older, disciplinary biopolitics and a newer, emergent biopolitics. The older biopolitics is a mode of governance – which often includes coercive techniques – exercised by sovereign states to regulate their populations’ health. The newer form, they argue, is a mode of ‘self-management’ or ‘somatic individuality’ that reflects the rising importance of a politics of risk and the ‘responsibilization’ of patient-consumers for their own health in ‘advanced, liberal societies.’ In a limited sense, the ‘older’ mode of biopolitics seems to be at work in Brazil's public health sector, where hormones are prescribed according to a logic of population control tied to national development (Sanabria 2010). The regulation of reproduction is here framed in terms of moral responsibility to the collectivity, with working class adolescent and young mothers who ‘fail’ to control fertility often shamed by Ob-Gyns (McCallum 2005, de Zordo 2012).

At first glance, the ‘newer’ self-management mode of biopolitics seems to be more clearly at work in the use of sex hormones in private practice medicine. Women often use hormones to manage affect or limit risks to sexual relationships due to partners’ straying by chemically boosting their desire with T. While in the private sector sex hormones are framed within a discourse of personal autonomy, freedom and choice, their actual use reflects differences between provider and client expectations as well as social pressures and constraints that problematize the notion of ‘self-management’ (Sanabria 2010). Class dynamics also shape the management of pre-menstrual tension, one of the key indications for menstrual suppressive contraceptives in Brazil. The addition of testosterone to implants is presented as enabling desire for busy middle- and upper-class Brazilian women. Among low-income patients who use methods such as *Depo-Provera* (also a menstrual suppressive), rationales have focused more on the possibilities this affords for work, where menstruation is seen as depleting strength. Across classes, women explained that they adopted menstrual suppressive treatments to manage their emotional oscillations, be these cyclical feelings of depression, irritation or anger, which impede domestic and work relations.

Like hormonal therapies, plastic surgery is practiced in both public and private healthcare in ways that reflect the class dynamics of Brazilian medicine. Once seen as the preserve of the elite, cosmetic surgery has been “democratized,” as one surgeon boasted. In the private sector the expansion of consumer credit and the taming of inflation brought surgery within reach of the middle class. Brazilian cities now have some of the highest concentrations of plastic surgeons in the world. The routinization of cosmetic surgery among wealthier Brazilians has contributed to a perception that the practice is an integral part of modern management regimes for women's health (Edmonds 2013b).

The aspirational aspect of demand for both *hormônio* and *plástica* is illustrated by the role that celebrity patients – and celebrity doctors – play in rendering these technologies socially visible. *Artistas* (celebrities) use cosmetic surgery to enlarge or lift their breasts or buttocks or narrow their waists and widen their hips. These uses have also been adopted by ‘ordinary’ consumers who learn about celebrity *plásticas* from magazines such as *Playboy* or *Plástica & Beleza* (*Plástica & Beauty*). Some surgeons complain about the unrealistic expectations of patients who walk into their office with a photo of an *artista*. Many *plástica* procedures aim at a kind of hyper-feminine ‘perfection’ linked to celebrity culture and Brazil's racialized national identity (Edmonds 2010).

Celebrities have played a key role in popularizing hormonal therapies as well. A cover story on how *telenovela* actress Carla Regina lost weight explains that her boyfriend, gynecologist Malcolm Montgomery, gave her tailored hormonal implants.[Fn en0007] Montgomery is well known as a gynecologist of the ‘stars.’ He explains that he switched ‘Carla's’ implants as the previous testosterone combination ‘injected protein into her muscles augmenting their volume. The new combination removes excess swelling and thins her silhouette.’ Montgomery added that ‘their prime medical function is to control hormonal fluctuations and reduce menstrual episodes,’ but ‘the specialist’ noted they also have ‘this interesting aesthetic effect.’

The ‘aesthetic effects’ of hormonal implants have been highlighted in mainstream news media. Coverage of Säo Paulo's Fashion Week 2012, for example, presented hormonal implants as *Chipes da beleza* (beauty chips) used by models to suppress menstruation, diminish cellulite, and avoid weight-gain. The only problem with the ‘hormonal chip,’ stated model Natália Zambiasi, is that ‘it makes you *tarada* (a sex maniac).’ Footballer Ronaldo's ex-girlfriend, model Raica Oliveira, explained she used the implant for eight years for aesthetic aims: ‘[Without it] I need to work out more, my bum is sagging, when I use the implant it is all firm and hard.’ Another model pointed out: ‘You “dry-out,” de-swell, lose weight and cellulite, gain muscle, your body becomes firmer and your skin texture improves. Plus you free yourself of menstruation!’ Celebrity gynecologists such as Montgomery and Elsimar Coutinho promote their work through ‘partnerships’ with the fashion industry. One modeling agency is reported to have given a model an advance payment of R$3500[Fn en0008] for a tailored hormonal treatment. While the Brazilian Society for Endocrinology and Metabolism and the Federal Council of Medicine have criticized such aesthetic indications, other medical societies actively promote implants while private clinics evade efforts at tighter regulation through legal loopholes.

These uses of *plástica and hormonio* may seem to represent a ‘new’ biopolitics described by Rose (2007) oriented towards risk management; however they are not only a means of working on the self or ‘somatic individuality.’ For one, they point to the importance of sexual and class aspirations and celebrity culture in Brazilian medicine. These practices also reveal the gaps between the vast possibilities that private healthcare affords and its uneven trickling-down into public health services, where care is often provided as a ‘favor’ or ‘charity’ rather than a ‘right’ (Sanabria 2010; Edmonds 2010). Medical techniques are thus adopted not only to constitute oneself as a ‘good’ citizen and *avoid* health risks; they involve *taking* risks to emulate sexual and aesthetic practices that are used by celebrities as routine self-care. Although these therapies are often practiced below the radar of state biopolitics, they are not therefore ‘individual’ or devoid of sociality. Self-management – or in the local idiom, *self-care* – is frequently about taking care of relationships in ways that do not fit notions of neoliberal ‘self-management.’ After all, *individualista* is a pejorative term. *Cuidar-se* is not (just) about the self. In the Brazilian cases we bring together here, it is also about making social relations through work on bodies. We now analyze how these techniques are embedded within shifting gender relations in Brazil to account for their local uses.

## The social dimensions of self-care: managing passage through the female life-course

A striking feature of both *plástica* and *hormônio* is that they are presented as technical means to improve one's quality of life. As the range of traits that can be rectified, modified or enhanced increases, the link between what *can* and what *should be* altered shifts. In his cultural history of testosterone, Hoberman (2005, p. 8) notes that a ‘drug-enhanced fitness’ made possible by the prescription of estrogens or androgens can be experienced as ‘a mandate to be sexually active even in the absence of desire’. This obligation to be both desirable and desiring fits with much of what we observed in our respective field sites.

We illustrate this point by analyzing how *plástica* and hormonal therapies manage passage through the life-course. The trajectory outlined below is a ‘therapeutic ideal type,’ not an official clinical recommendation. Doctors and patients choose from among these therapeutic possibilities, and it would be unlikely for them to all be adopted by one person. Our aim is to show how women use these techniques to respond to social and somatic expectations surrounding passage through the life course.

### Onset of menses

Some plastic surgeons hesitate to operate on teenagers, who are assumed to be less emotionally stable than adults. However, one surgeon argued that ‘nothing prevents adolescents from seeking a new appearance.’[Fn en0009] *Plásticas* are in some instances even given to teenagers for their *festa de quinze anos* (15th birthday celebrations). One woman Sanabria interviewed explained the pressure her adolescent daughter felt to ‘maintain appearances’ as her school friends were being ‘bought a pair of breasts’ or a ‘new bum’ by their parents.

Hormonal contraceptives may be prescribed prior to the onset of regular menstruation, to delay menarche, manage irregular bleeding (common in early years) or acne. Gynecologist Coutinho controversially proposed administering *Depo-Provera* on mass to low-income teenage girls to limit teen pregnancies. The campaign poster showed an image of a perfectly toned *mestiço* girl's belly with bellybutton ring, enticing young girls to opt for the hormonal methods on offer and keep a nice flat belly instead of acquiring a flaccid, post-natal one.[Fn en0010]

### Pregnancy and childbirth

Pregnancy and childbirth are seen to pose risks and cause iatrogenic damage to the female body, which can be ‘fixed’ through surgical intervention.[Fn en0011] A range of techniques are available to meet the norm imposed by star culture – as well as medical culture – to rapidly return to ‘shape’ post-partum.[Fn en0012] Post-partum *plásticas* include breast and abdominal surgeries as well as liposuction and *lipoescultura*, which redistributes fat in order to ‘contour’ the body's silhouette. Injunctions to manage weight-gain during pregnancy mix aesthetic concerns with health risks concerning gestational diabetes or high blood pressure. *Plástica* is also integrated with mainstream Ob-Gyn, with Ob-Gyns referring mothers to plastic surgeons who promise to return the belly, vagina or breasts to their pre-pregnancy condition. In Brazil there are high rates not just of cosmetic surgery but also surgeries such as tubal ligation, episiotomy, hysterectomy and C-section (see Araujo and Aquino 2003; Giffin et al. 2003; McCallum 2005 and Edmonds 2013a for a discussion of the links between these surgeries). There are complex causes behind Brazil's high rate of C-sections, including physician convenience, but the surgery is sometimes represented as a legitimate means to avoid damaging the perineum and compromising future sexual well-being (Carranza 1994; Behague 2002).

However, this goal coexists with decidedly ‘aesthetic’ practices or ones aimed at male partners. Vaginal births routinely include episiotomies followed by the *ponto de marido*, the extra ‘husband's stitch’ (Diniz and Chacham 2004). In public hospitals Sanabria (2011) found that some women unable to ‘acquire’ a C-section are given post-partum vaginal *plástica*. In the private sector ‘intimate *plásticas*’ are marketed to increase sexual pleasure and improve genital appearance. And while C-sections avoid pelvic damage, they create aesthetic damage to the abdomen – scars and flaccidity – that can be repaired with *plástica*. The routinization of the C-section thus contributes to a new medical ‘need’ for corrective aesthetic surgery (Edmonds 2013a).

### Breast feeding

Breastfeeding is widely said to cause aesthetic damage as well as interfere with women's sexual desire and availability to their partners. One of the most popular surgeries in Brazil is breast reduction, which unlike in much of Euro-America, can have a primarily aesthetic rationale. The surgery is said by surgeons and patients to emulate a national corporeal ideal – often seen as an Afro-Brazilian legacy – that emphasizes slender waists, full hips, thighs and buttocks, and relatively small breasts (Hanchard 1999). But breast surgery is also positioned as a means to manage an erotic body threatened by reproduction (Edmonds 2013b).

### Menopause

Hormonal replacement therapy (HRT) is widely prescribed in Brazil for multiple, often blurred aims: controlling menopausal symptoms, treating sexual dysfunction or boosting sexual experience. The mainstream news media refers to the inclusion of testosterone in HRT as the ‘whipped cream of hormonal repositioning.’ Treatments include implants containing testosterone as well as T-gels, widely available in compounding pharmacies to be applied topically.

Many Brazilian gynecologists dismiss the health risks of HRT that were shown in studies by the Women's Health Initiative (WHI). One private-practice gynecologist who renewed a HRT prescription for the 12th consecutive year was somewhat naively asked by the ethnographer about the medical rationale for such lengthy treatment. He angrily shrugged off the WHI studies as the product of ‘excited North American hairy feminists.’ ‘My patients,’ he added, ‘aren't concerned about future risks, they just want to stay beautiful and maintain their sexuality, why should I stop them?’

Sanabria often heard gynecologists present HRT as a form of basic self-care. With menopause, ‘the body’ – one gynecologist told her – ‘begins to age and decay. Women today live longer and stay sexually active. HRT just helps them keep up with their lives and maintain their relationships.’ Implicit is the idea that a man – whose sexuality is naturalized – is entirely justified to seek sex from younger women if his wife becomes *largada* (unkept).

Zunara is 61 and has always refused HRT. A lesbian, feminist and medical doctor, she is outraged that *hormônio* is so prevalent in Brazilian gynecology. During a consultation, her doctor expressed shock at how *resecada* (dry) Zunara's vagina was and started to instill fear in her, speaking of ‘a scorched field’ and risks of infection. Reflecting on the event, she concluded that gynecologists who are ‘ferocious *hormônio* prescribers’ are used to ‘nice pink vaginas’ and not to seeing ‘what a 61 year old vagina *really* looks like.’ The norm, by virtue of pharmaceutical intervention, has changed. It follows that 61-year old vaginas are no longer, in private practice, ‘dry.’ What is a feminist response to this, Zunara wondered? Promoting sexual pleasure beyond reproduction can be seen as part of a project of sexual empowerment. However, this seems to be happening through the imposition of a norm obliging women to remain sexually available and attractive according to an idealized representation of (youthful) femininity. Women like Zunara who opt out of HRT are considered ‘brave’, ‘alternative’ or irresponsible, and lacking in basic self-care.

Similarly, some plastic surgery patients described themselves or others as lacking sufficient *vaidade* (vanity), which in Brazilian-Portuguese often has positive connotations. Surgeons say *plástica* boosts self-esteem during menopause, presented as a difficult time for women. Márcia, who had been waiting for ten months to have a second facelift, discussed *plástica* in relation to the lifecycle: ‘When I began menopause three years ago, everything fell, fell really fast. Women age more quickly… menopause, hormones, breast feeding, giving birth, everything deforms the stomach, your body… and domestic life too is wear and tear, stress. And also men demand more of us.’ For Márcia *plástica* is a means of compensating the damage due to physiological processes.

But Márcia's commentary also points to changing gendered sexual expectations in middle age. Brazilian aging norms have changed rapidly since the 1980s. As in much of the industrialized world, Brazilian consumers have been ‘responsibilized’ for their health as new ideals of ‘successful aging’ have been popularized in the media (Debert 2010; Leibing 2005; Edmonds 2014). These norms include a striking focus on managing sexual fitness and beauty. News media promote an ‘arsenal’ of medical and cosmetic practices aimed at ‘sex after 40’: from sex toys and psychotherapy to testosterone-enhanced hormonal regimes and cosmetic surgery.

Marketing discourse, however, does not simply create *ex-nihilo* new medical needs, as has been argued by some critics of medical enhancement. In this view, which resembles critiques of irrational consumption, increasingly powerful medical institutions create new market niches for previously unnecessary technologies. Yet *hormônio* and *plástica* have been enthusiastically embraced partly because they ‘fit’ long-standing medical and social realities; they have become, we suggest, integrated into familial and sexual life. In Brazil, ‘recreational’ or ‘plastic’ sexuality (Giddens 1993) coexists with ‘traditional’ forms of patriarchal sexuality; sexually open youth cultures thrive alongside the dissemination of the ‘modern’ norm of romantic, companionate marriage (Rebhun 1999; Mayblin 2010). The men and women we met often saw sexual relationships as highly competitive (Goldenberg 2010). While official justifications of plastic surgery often stress its psychological benefits, in intimate conversations concerns about maintaining relationships were a major theme. Several women had undergone *plástica* following a break-up. One recently divorced young mother received Sanabria in her home in her postoperative garter for an interview on her use of menstrual suppressive implants. She explained that she had always wanted to have *plástica* but that her ex-husband had not supported the idea; she now wanted to show the world she could bounce back from a humiliating divorce. *Plástica* and hormones are often used to maintain different types of relationships – domestic, professional, class – in highly uncertain environments. Market niches for medical technology coexist with what might be called social niches created by changing sexual and gender norms and aspirations.

Paul Rabinow's (1996) discussion of biosociality emphasizes the ‘horizontal’ relationships that form between people in shared genetic, risk or disease categories. It emphasized how some patient groups have challenged older lay-expert relationships. What this argument overlooks are the fracture lines in social groups formed around disease or risk, the places where the collective comes apart at the seams. In Brazil, elite modes of medical consumption often become models for the popular classes. There is a powerful aspirational dimension to entering the biosocial which is not always realized, perhaps doomed by capitalist logics to remain incomplete. The bio in ‘biosociality’ is thus also becoming – not something given or foundational to identity in the sense that genetic or disease classifications are at times understood by Euro-American publics (Strathern 1992).

## Conclusion

This paper has brought together materials from our respective research sites to explore areas of clinical and social overlap in the uses of *plástica* and *hormônio*. While there are important differences between these therapies, we have sought to show that together, they are embroiled in redefinitions of normal femininity and caught up in complex class processes. We have argued that they are used in highly experimental ways that blur the boundaries between ‘well’ and ‘better than well.’

These uses have different – in a sense ‘inverted’ – clinical logics. Sex hormones are a ‘normal’ part of reproductive healthcare. Yet they are also used for medical and social purposes that go beyond this indication: from *travestis*’ erotic body sculpting and menstrual suppression to the aesthetic effects of the ‘beauty chip’ and sexual self-care. On the other hand, cosmetic surgery is not ‘officially’ a part of reproductive healthcare. Yet it is being integrated with obstetrics and gynecology, practiced in a public healthcare system, and used to correct iatrogenic damage resulting from mainstream women's healthcare. As such it ‘borrows’ legitimacy from the clinical contexts in which it is practiced, as a routine aspect of psychological, reproductive and sexual management. One result is that ‘aesthetics’ is emerging as a key dimension of women's routine healthcare in Brazil (Edmonds 2013a).

We aimed to expand the focus of a bioethics literature concerned with utopian or dystopian technologies to include their ordinary, everyday uses in Brazilian medical institutions. This is an important critical task due to the sheer prevalence of the therapies we discussed. With the growth of ‘drugs-for-pleasure’ (Race 2009), anabolic steroid use, recreational Viagra, neuro-enhancement and other experimental means for boosting and coping, enhancement has become a crucial health and ethical issue ‘beyond the West.’ This point is important as critiques of medical enhancement often assume that it spreads from a Western ‘ground zero’ to other places. We argue that this timeline overlooks global flows and feedback between regions. Brazilian experiments with sexual and aesthetic medicine are ‘exported’ to richer, Western countries. Italian medical tourists travel to Brazil for cosmetic surgery not because it is cheaper, but because it is more glamorous. Brazilian doctors such as Elsimar Coutinho play a pivotal role in producing clinical ‘evidence’ in support of a global menstrual suppression discourse and off-label uses of sex hormones.

These developments entail considerable risks.[Fn en0013] As technologies become embedded in routine healthcare they also become more medically acceptable; health risks hence become easier to minimize or dismiss. But these therapies are becoming routinized not only because they have been aggressively or cynically marketed. Rather, they reveal conflicts in gendered norms concerning how to be properly and ideally desirable and desiring; how to balance reproductive and sexual desires; and how to be a ‘good’ mother and a ‘modern’ woman.

## Notes

1. Our comparative analysis was not planned before we began fieldwork, but was initiated after conversations where we perceived several important overlapping themes in our respective research.
2. We use the term ‘therapy’ with implied quotation marks that we omit for simplicity: that is, we do not imply surgery or sex hormones are always therapeutic.
3. Technologies used to deliver hormones have also changed significantly. The 21/7 regimen was adopted to reproduce a monthly artificial bleeding episode that mimicked a ‘natural cycle’ (Marks 2001; Kissling 2013; Mamo & Fosket 2009). The stabilization of a normal 28-day cycle was only later written into the design of the object itself (Sanabria 2009).
4. Elsimar Coutinho, controversial Bahian gynecologist and author of *Is Menstruation Obsolete?* in whose clinic Sanabria carried out ethnographic work, claims to have discovered the contraceptive effects of medroxyprogesterone acetate (in *Depo-Provera*) and has developed a range of hormonal implants in Brazil.
5. These occupy roughly 10% of the pharmaceutical market according to some estimates and can be found in most shopping malls (Sanabria 2009).
6. Cosmetic procedures are also (legally) performed by doctors without training in plastic surgery. One patient alleged that obstetricians had, in the 1980s, performed liposuction after delivering a baby as a ‘favor’ to the patient (a claim surgeons dismissed).
7. 
http://boaforma.abril.com.br/famosas/famosas-em-boa-forma/carla-regina-emagreceu-5-kg-532732.shtml (consulted 17 October 2013).
8. Roughly €1185.
9. ‘Reino das Formas Perfeitas,’ *Época*, 19 June 2000.
10. This campaign poster was brought to our attention by Silvia de Zordo.
11. 
*Hormônio* and *plástica* are often used to correct iatrogenic damage caused by other routine medical technologies. *Plástica* is used to correct damage from mastectomy, previous *plásticas*, as well as C-sections or assisted vaginal deliveries. Hormonal combination drugs that have testosterone can optimize functioning harmed by other hormonal therapies or pharmaceuticals, or else replace endogenous hormones lost through hysterectomy.
12. This is not specific to Brazil, as attested to by the media hype surrounding Kate Middleton's exhibition of her flat and toned belly less than three months after giving birth to baby Prince George.
13. For more discussion of these risks see Edmonds (2013a) and Sanabria (2010).
